# CH_3_OH^•+^ + CH_4_ Reaction: Mechanistic Insights and Reaction Rates for Astrochemical and Atmospheric Environments

**DOI:** 10.3390/molecules30051029

**Published:** 2025-02-24

**Authors:** Mauro Satta, Daniele Catone, Mattea Carmen Castrovilli, Francesca Nicolanti, Antonella Cartoni

**Affiliations:** 1 Institute of Nanostructured Materials—CNR (ISMN-CNR), Department of Chemistry, Sapienza University of Rome, P. le Aldo Moro 5, 00185 Rome, Italy; 2 Istituto di Struttura della Materia—CNR (ISM-CNR), Area della Ricerca di Tor Vergata, Via del Fosso del Cavaliere, 00133 Rome, Italy; daniele.catone@cnr.it; 3 Istituto di Struttura della Materia—CNR (ISM-CNR), Area della Ricerca di Roma 1, Via Salaria km 29.300, 00015 Monterotondo, Italy; matteacarmen.castrovilli@cnr.it; 4 Department of Physics, Sapienza University of Rome, P. le Aldo Moro 5, 00185 Rome, Italy; francesca.nicolanti@uniroma1.it; 5 Department of Chemistry, Sapienza University of Rome, P. le Aldo Moro 5, 00185 Rome, Italy

**Keywords:** ion chemistry, methanol radical cation, synchrotron radiation, methane activation, canonical rate, microcanonical rate

## Abstract

The reaction between methanol radical cations and methane, producing methyl radicals and protonated methanol, is pivotal to both astrochemical and atmospheric processes. Methanol and methane are the most abundant organic molecules in space and Earth’s atmosphere and central to molecular synthesis under different environmental conditions. Here, we present a combined experimental and theoretical investigation of the ion–molecule reaction between CH_3_OH^•+^ and CH_4_. The study explores the reaction mechanism and energetics under ionized conditions utilizing quantum chemical methods and experimental data. The findings reveal that the reaction’s non-thermal behavior becomes pronounced when CH_3_OH^•+^ is vibrationally excited by photon absorption above the ionization threshold, as can happen in the presence of ionizing agents like cosmic rays. Conversely, in thermal equilibrium conditions, the reaction accelerates as temperatures decrease, as suggested by canonical rate coefficient calculations. The products can initiate further chemical reactions, shaping molecular networks in the interstellar medium and affecting atmospheric trace gas balances.

## 1. Introduction

The reaction between methanol radical cations and methane, producing methyl radicals and protonated methanol, has significant implications in both astrochemical and atmospheric contexts. Astrochemistry highlights how gas-phase reactions drive the formation and destruction of molecules across the universe, especially in environments like planetary atmospheres and the interstellar medium (ISM). Space acts as a giant chemical reactor where high-energy radiation ionizes and dissociates simple molecules, generating reactive ions and radicals. These intermediates generally participate in barrier-free, highly exothermic reactions, leading to complex molecular synthesis. Methanol, one of the most abundant organic molecules in space, was first observed in 1970 [1] and has been detected across ISM regions, including hot cores and dark clouds [2,3,4,5]. The methanol radical cation (CH_3_OH^•+^) is likely present in the ISM but still undetected, probably due to its high reactivity. Its existence could be crucial for synthesizing complex organic molecules, providing insight into the chemistry behind life’s building blocks and interstellar molecular networks. Similarly, methane is a key astrochemical molecule, and a simple hydrocarbon widely found in space. Its presence has been observed on Earth, Mars, and icy bodies like Triton and Pluto. Within the ISM, methane accumulates on dust grains, forming icy mantles that serve as micro-reactors for molecular synthesis [6]. These icy particles are central to interstellar chemistry, driving chemical evolution and contributing to complex organic formation in space. The methyl radical CH_3_ is an essential intermediate in the ISM’s ion–molecule chemistry [7]. The formation of CH_3_ occurs through various reactions, including the photodissociation of methane while protonated methanol is implicated in forming interstellar dimethyl ether, which arises from interactions between methanol and its protonated form through energetically favorable pathways. Dimethyl ether’s formation is further facilitated by reactions transferring protons to ammonia, spurring additional interstellar reactions [8].

Methanol and methane are also relevant for atmospheric chemistry. Methane, as a potent greenhouse gas, has contributed about 0.6 °C to global warming since pre-industrial times [9]. It is emitted both naturally (e.g., from wetlands) and anthropogenically (e.g., agriculture and fossil fuel combustion). Methane’s atmospheric lifespan is around nine years, primarily limited by oxidation via hydroxyl radicals [10]. Reducing methane emissions is considered an effective short-term strategy for mitigating global warming [11]. Methane has fluctuated in growth rate, with causes not fully understood. Probably its sources and the variability of its primary sink, the hydroxyl radical, partially explain the global methane budget changes [12,13]. However, other processes may take place, considering also that the reaction of methane with OH is very slow about 6.59 × 10^−15^ cm^3^ molecule^−1^ s^−1^ at 298 K [14] and the reasons for the pause in growth in 1999 and renewed growth from 2007 remain quite unclear. As for the methyl radical, although present in very low tropospheric concentrations, it serves as a significant short-lived species with a lifespan of seconds to minutes [15]. Methyl radicals are mainly produced in the atmosphere via the photolysis of volatile organic compounds (VOCs), mainly methane, which is identified as the largest natural source of CH_3_ [16]. Once formed, the methyl radical can react with several atmospheric species, including oxygen, nitrogen oxide, and ozone. Notable reactions involving CH_3_ in the troposphere include its reaction with oxygen to form CH_3_O_2_ [17], with nitrogen oxide to yield HCN and H_2_O [18], with hydroperoxyl radical (HO_2_) [19] to form various products, and with ozone in a reaction contributing to ozone depletion [20]. Methanol emissions [21], primarily from land plants [22], contribute up to 350 teragrams annually [23], making up about 20% of global VOC emissions, second only to methane [24]. Methanol removal occurs through surface deposition, ocean absorption, and reactions with hydroxyl radicals [25,26,27], and its photochemistry is closely connected to ozone and hydroxyl radical levels, highlighting methanol’s role in atmospheric dynamics [28] since it directly and indirectly influences the abundances of many other tropospheric trace gases. Protonated methanol has also been observed in lower atmospheric layers [29].

Given their shared relevance in both space and Earth environments, the reaction between methanol and methane in neutral and ionized environments (thunder, lightning, radiation etc.) remains an essential subject of study for understanding molecular interactions across vastly different settings. In this work, we report a joint experimental and theoretical investigation of the ion–molecule reaction between methanol radical cation CH_3_OH^•+^ and CH_4_ leading to methyl radical CH_3_ and protonated methanol CH_3_OH_2_^+^, which are relevant prebiotic species. The research aims to provide insights into how environmental conditions and energetics may influence the title reaction. The paper is structured as follows: Section 2 presents the materials and methods, detailing both experimental and theoretical approaches; Section 3 covers the results and discussion; and Section 4 provides the conclusions. For the sake of clarity, the dot • in CH_3_OH^•+^ will be omitted throughout the manuscript.

## 2. Materials and Methods

### 2.1. Synchrotron Experiments

The experiments were conducted at the CiPo (Circular Polarization) beamline at ELETTRA, following the setup and procedures detailed in our previous publications [30,31,32]. In summary, radiation was produced by an electromagnetic elliptical undulator/wiggler and monochromatized using a Normal Incidence Monochromator (NIM) within the vacuum ultraviolet (VUV) energy range of 8–40 eV. An aluminum grating was used to restrict operations to an energy range of 8–17 eV, with a resolving power of approximately 1000. Photon energy calibration was performed using autoionization features in the total photoionization spectrum of argon, focusing on the 3p spin-orbit components. To eliminate higher-order radiation below 11.7 eV, a lithium fluoride (LiF) filter was employed. Methanol was introduced into the ion source through a leak valve, maintaining a pressure between 10^−6^ to 10^−5^ mbar. In the synchrotron experiments, the methanol radical cation was produced by ionizing methanol in the ion source at a pressure of approximately 10^−5^ mbar, using synchrotron radiation at fixed or variable photon energies from 10.85 to 11.65 eV. This energy range was chosen because the ionization energy of methanol is 10.84 ± 0.01 eV [14] and to prevent the fragmentation of CH_3_OH^+^ into CH_2_OH^+^, which occurs at 11.649 ± 0.003 eV [33]. Moreover, the formation of CH_2_O(H)H^+^, more stable than isomer CH_3_OH^+^ in its ionic ground state by about 45.0 kJ/mol [34], was excluded since the isomerization barrier from CH_3_OH^+^ to CH_2_O(H)H^+^ is very high, 104.6 kJ/mol, and not accessible in our experimental conditions [35]. Ions generated in the source were directed into the reaction cell (octupole) with nominal collision energy (CE) of 0 eV and an energy spread of approximately 120 meV. The neutral reagent CH_4_/CD_4_ was introduced into the octupole (reaction cell) at various nominal pressures, ranging from about 10^−5^ to 10^−4^ mbar at room temperature. Mass spectra were recorded before and after the reaction of methanol radical cation with methane at an arbitrary chosen photon energy of 11.5 eV within the mass range 10 < *m*/*z* < 40, with a collection time of 4 s per point at the nominal methane pressure of 9.5 × 10^−5^ mbar. Intensities of reactant and product ions from the ion–molecule reactions between CH_3_OH^+^ and CH_4_ were collected across an energy range of 10.85 < *hν* < 11.65 eV with energy steps of 0.05 eV, acquisition time of 30 s per point, and at nominal methane pressures of 5.5, 7.5 and 13 × 10^−5^ mbar to verify the linear relationship between product/reagent ratio and pressure at different photon energies.

CH_3_OH and CD_4_ were obtained from Sigma-Aldrich (Milan, Italy) and used at room temperature as supplied. The CH_3_OH had an HPLC gradient grade purity of 99.93% and CD_4_ had 99 atom % D. CH_4_ was purchased from SIAD(Trieste, Italy), with a purity >99%.

### 2.2. Computational Details

The energetic and dynamic characterization of the title reaction was conducted using Density Functional Theory (DFT) [36] as the framework, enabling the exploration of the potential energy surface (PES) associated with hydrogen transfer reaction. In particular, the ab-initio calculations used the Density Functional Theory double-hybrid formalism [37], which describes the radical nature of the molecules reacting during the hydrogen transfer processes. We calculated the reaction enthalpy at various theoretical levels, specifically MP2, B3LYP, B2PLYP and M06-2X, using different basis sets: aug-cc-pVDZ, cc-pVTZ, and aug-cc-pVTZ. Ultimately, B2PLYP/aug-cc-pVTZ [37] was selected due to its good agreement, within 2 kJ/mol, with the experimental reaction enthalpy reported in the literature. The reactive region of the potential energy surface (PES) was mapped by fully optimizing all degrees of freedom except those used to track the reaction, as defined in Section 3. The distances chosen to follow the reaction were scanned with a variable step size, with a minimum increment of 0.01 Å. These ab-initio calculations determined the Minimum Energy Path (MEP), useful for computing reaction rate coefficients. The kinetics of the reaction were modeled using the variational transition state theory (VTST) approach, specifically designed for barrierless reactions [38]. Zero-point energy corrections were applied to all computations, with harmonic vibrational frequencies scaled by a factor of 0.986 [39]. Charge and spin densities were evaluated using Natural Population Analysis [40]. The MEP served as the basis for calculating the total molecular partition functions, *Q(T)*, of the reactive complex over a temperature range of 200–3000 K. These partition functions, integral to VTST, were employed to identify the kinetic bottleneck in the reactive flux along the MEP [41]. All quantum chemical computations were performed with the Gaussian09 software package [42].

## 3. Results and Discussion

**Figure 1:**
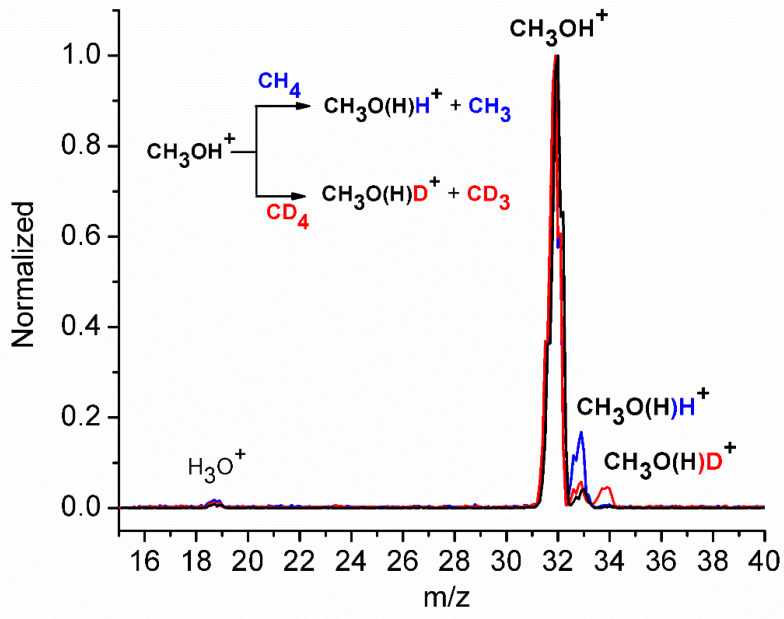
Mass spectra of the CH_3_OH^+^ ion acquired at the photon energy of 11.5 eV without (black line) and with CH_4_ (blue line) and CD_4_ (red line) in the reaction cell, at the nominal methane pressure of about 9.5 × 10^−5^ mbar and CE = 0. In the inset, the reaction scheme.

Initially, after the formation of the methanol radical cation in the ion source, mass spectra were acquired in the absence of methane in the reaction cell (octupole), confirming the presence of only methanol in the apparatus (signal at *m*/*z* = 32), with only minor unavoidable traces of water, which were protonated to form H_3_O^+^ (*m*/*z* = 19) by methanol ions (black line in Figure 1). The signal at *m*/*z* = 33 corresponds mainly to the isotopic contribution of ^13^C in methanol and to protonated CH_3_OH_2_^+^ formed in the ion source [43,44].

**Figure 2:**
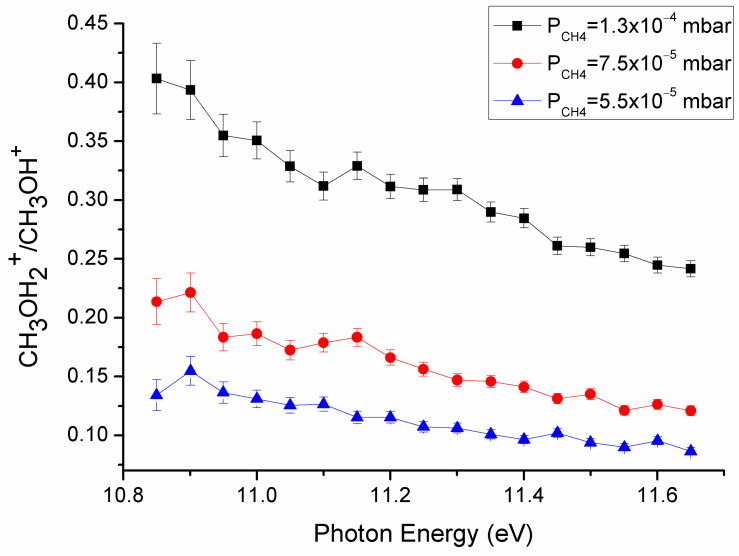
CH_3_OH_2_^+^ (*m*/*z*= 33)/CH_3_OH^+^ (*m*/*z* = 32) ratio as a function of photon energy (eV) at different nominal methane pressures.

Subsequently, methane was introduced into the cell at a pressure of approximately 9.5 × 10^−5^ mbar, and all the ionic products were detected using the quadrupole analyzer. The data for the ion–molecule reaction are shown in Figure 1 (blue line and inset), where an increase in the peak at *m*/*z* = 33 was observed, compared to the spectrum acquired without methane in the cell (black line, Figure 1). To confirm that the reaction involves a hydrogen atom transfer (HAT) from methane to the methanol radical cation, the reaction was also performed with CD_4_, where a peak at *m*/*z* = 34, corresponding to CH_3_O(H)D^+^ formation, was observed (red line in Figure 1 and inset). This finding definitively confirms the HAT between CH_3_OH^+^ and CH_4_/CD_4_, leading to the production of CH_3_/CD_3_ radicals. The reaction was also performed with CD_3_OH^+^ (*m*/*z* = 35) and CH_4_ or CD_4_ inside the cell, where peaks at *m*/*z* = 36 (CD_3_OH_2_^+^) and *m*/*z* = 37 (CD_3_O(H)D^+^) were detected, respectively. The reactivity was also studied as a function of photon energy used to produce CH_3_OH^+^, and at different nominal CH_4_ pressures ranging from 5.5 × 10^−5^ mbar to 1.3 × 10^−4^ mbar. The ratio of the intensity of the product (CH_3_OH_2_^+^, *m*/*z* = 33) to the reagent CH_3_OH^+^ (*m*/*z* = 32) is reported in Figure 2.

As shown, the efficiency of the reaction decreased with photon energy, as already observed in different fast exothermic reactions [45,46]. From the thermochemical database [47] we calculated, for this reaction, an exothermicity of −50.7 kJ/mol at 298 K, demonstrating once again how the HAT from CH_4_ is often thermochemically favored and observed when a gas phase reaction involves radical cations [48]. Moreover, the reactivity increased with the methane pressure, as expected. To gain a deeper understanding of the reaction dynamics, ab initio theoretical calculations were carried out.

**Figure 3:**
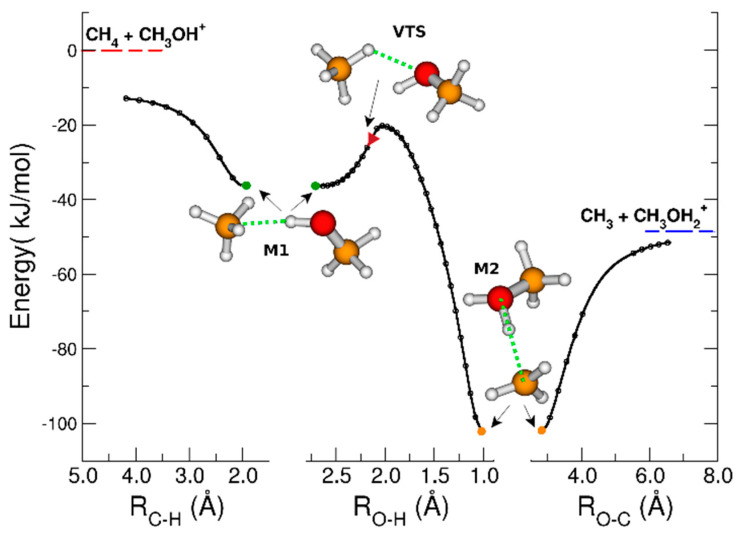
Minimum Energy Path of the reaction between methanol radical cation and methane. The green circles indicate the position of the local energy minimum M1, the red triangle the position of VTS and the orange circles the position of the absolute minimum M2. The red line in the first panel is the energy reference associated with the reactive molecules, while the blue line indicates the energy of the products. See the text for further details.

In Figure 3, the Minimum Energy Path (MEP) is illustrated, taking into account different reaction coordinates: R_C-H_ (left panel), which represents the distance between the carbon of methane and the hydrogen bound to the oxygen of methanol; R_O-H_ (central panel), the distance between the hydrogen of methane and the oxygen of methanol; and R_O-C_ (right panel), the distance between the carbon of methane and the oxygen of methanol.

**Figure 4:**
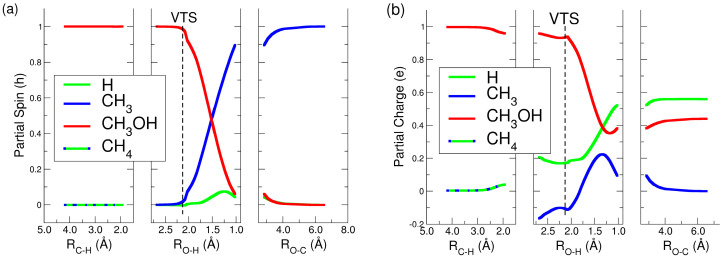
(**a**) Partial spin distribution of the reactive species along the MEP. (**b**) Partial charges of the reactive species along the MEP. H, presented as the green line, is the hydrogen transferred from methane to the oxygen of methanol.

Along the MEP, two energy minima, labeled M1 and M2, were identified on either side of the variational transition state (VTS) [38]. The energies of M1 and M2 were −36.3 kJ/mol and −101.9 kJ/mol lower than those of the reactants, respectively. The VTS, denoted by a red triangle, was located near a sub-barrier at −23.5 kJ/mol. At this point, the O-H bond was not yet formed (2.13 Å), and the system must overcome the sub-barrier located at −20.2 kJ/mol to reach M2, where hydrogen binds more closely to oxygen (1.02 Å). The overall process released 48.6 kJ/mol, aligning closely with the calculated literature value of 50.7 kJ/mol [47]. Figure 4a, b presents the results regarding spin and charge distributions along the three analyzed coordinates.

Along the first coordinate (R_C-H_), spin remained localized on methanol (red line), while methane exhibited a small charge around 0.04 e, represented by a mix of green and blue lines. In the second coordinate (R_O-H_), both charge and spin remained stable up to the VTS structure, where the distance between methane’s hydrogen and methanol’s oxygen measured 2.13 Å. As the hydrogen from methane (green line) approached the oxygen in methanol, the spin of CH_3_OH^+^ declined rapidly, shifting to the forming CH_3_ group up to (blue line) 1.0 *ħ* in the right panel of Figure 4a, where the products were formed. Concurrently, the charge decreased in CH_3_OH^+^ and increased in both CH_3_ and the hydrogen detaching from methane (green line) until the O-H distance reached 1.17 Å, after which the charge stabilized in CH_3_OH^+^, increased in H, and declined in CH_3_ (left panel, blue line in Figure 4b). This picture clearly shows that charge and spin were redistributed after the formation of the VTS, that is, the bottleneck of the reaction.

**Figure 5:**
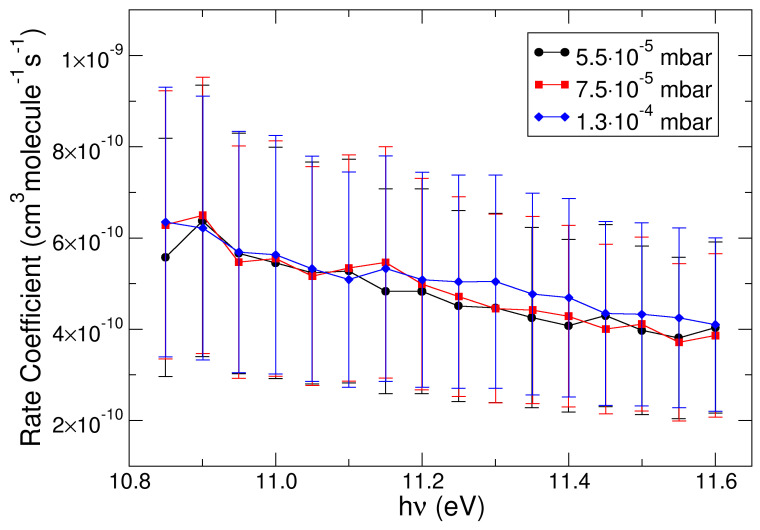
Rate coefficients for the title reaction as a function of the photon energy *hν* used to ionize the methanol molecule. The black dots, red squares, and blue diamonds represent rate coefficients at different methane pressures.

As regards the kinetic aspects of this reaction, the rate coefficients were derived from the experimental mass ratio data using the equation derived in our previous work [49]:

(1) $$\;k\left(hv\right)=\frac{\ln\left(1+R\left(hv\right)\right)}{\tau_{R}N_{CH_{4}}}\;\;\;\;$$

where *k*(*hν*) is the rate coefficient depending on the energy *hν* used to ionize the methanol molecule; *R(hν)* is the experimental product/reagent mass ratio reported in Figure 2; *N_CH4_* is the number density of the methane, which depends on its pressure (whose error is estimated as 20%); *τ_R_* is the characteristic time interval, which is assumed to depend on the nature of the ionized molecule as well as on the experimental setup, with a value of (1.7 ± 0.6) × 10^−4^ s determined from a previous work [44]. The experimental rate coefficients derived from Equation (1) are reported in Figure 5 for various CH_4_ pressures.

The rate coefficients had very similar values for the different CH_4_ pressures within the interval (0.55–1.30) × 10^−4^ mbar. The reaction was slower when the ionization photon energy *hν* was increased to 11.6 eV. These rate coefficients are below the Langevin rate of 1.12 × 10^−9^ cm^3^ molecule^−1^ s^−1^, indicating that the reaction efficiency was about 50% at the ionization threshold (*hν* = 10.85 eV) and it decreased to about 35% at *hν* = 11.60 eV.

**Figure 6:**
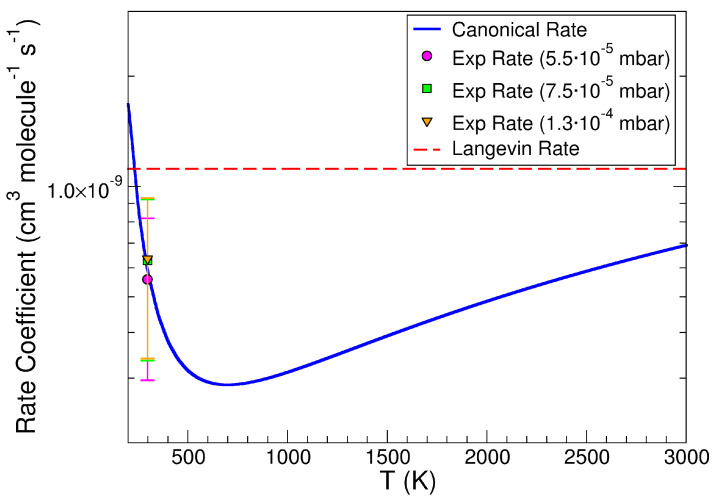
Canonical rate coefficients of the title reaction as a function of temperature. The purple circle, the green square, and the orange triangle represent the experimental rate coefficients at different pressures of CH_4_ at the ionization threshold *hν* = 11.85 eV. The dashed red line is the Langevin rate coefficient.

To gain insights into the microscopic mechanisms that control the reaction, the canonical VTS rate coefficients were calculated and are reported in Figure 6.

The agreement of the calculated canonical rate coefficient 5.81 × 10^−10^ cm^3^ molecule^−1^ s^−1^ at T = 300 K with the experimental rate coefficient at the ionization threshold is quite good, supporting the reliability of both the ab-initio data and the VTS complex calculations. The V shape of this rate coefficient as a function of temperature is indicative of the dual nature of this reaction. At low temperatures up to about 700 K, the reaction slowed down as the temperature increased, whereas from 700 K upwards the reaction sped up with the increase in *T*. The behavior of the rate coefficient in these two different temperature regimes can be discussed in terms of molecular partition function of the reagents and of the VTS complex. Under the low *T* regime, the molecular partition function of the VTS increased more slowly with *T* than the molecular partition function of the reagents; on the contrary, under the high *T* regime the opposite behavior occurred.

In Table 1, the parameters for the fitting of the experimental rate coefficients as a function of the photon energy in Figure 5 (P_CH4_ = 5.5 × 10^−5^ mbar) and of the canonical rate as a function of temperature in Figure 6 are reported.

The dependence of the rate coefficient on the energy of the ionizing photon was obtained by using the microcanonical approach with the hypotheses that the rotational and translational modes of the methanol cation and VTS complex were: (i) uncoupled with their internal vibrational degrees of freedom, and (ii) associated with a Boltzmann distribution at *T* = 300 K. The energy of CH_4_ was also characterized by a Boltzmann distribution at *T* = 300 K for both the internal modes, and the translational degrees of freedom. Hence, the microcanonical rate coefficient is given by:

(2) $$k_{µ}\left(E\right)=\sigma \frac{Q_{µVTS}^{ro}\left(300K\right)}{hZ\left(300K\right)Q_{CH_{4}}^{ro}\left(300K\right)Q_{CH3OH^{+}}^{ro}\left(300K\right)Q_{CH_{4}}^{vib}\left(300K\right)}\frac{N_{µVTS}\left(E_{reac}^{therm}+xE_{µVTS}+E_{µVTS}^{vib}\left(hv\right)\right)}{\rho_{CH3OH^{+}}^{vib}\left(E_{CH3OH^{+}}^{therm}+E_{CH3OH^{+}}^{vib}\left(hv\right)\right)}$$

where *E_µVTS_* is the energy difference between microcanonical variational transition state µ*VTS* and the reagents, whose geometry was calculated minimizing the vibrational number of states *N_µVTS_*, neglecting the vibrational frequency associated with the reaction coordinate of the reactive complex computed using the direct count Beyer–Swinehart algorithm [50]. The same algorithm was used to calculate the vibrational density of states of the CH_3_OH^+^ ion, $\rho_{CH3OH+}^{vib}$. The $Z\left(T\right)=\frac{{\left(2\pi µK_{b}T\right)}^{3/2}}{h^{3}}\;$function considered the relative translation density of states per unit volume averaged over a Boltzmann thermal distribution at *T* = 300 K. $Q_{µVTS}^{ro}\left(300K\right)$, $Q_{CH3OH^{+}}^{ro}\left(300K\right)$, $Q_{CH_{4}}^{ro}\left(300K\right),$ $Q_{CH_{4}}^{vib}\left(300K\right)\;$are respectively the molecular partition functions for the rotational modes of the µVTS and CH_3_OH^+^ and the rotational and vibrational modes of CH_4_. σ is the symmetry factor for the reaction. $E_{reac}^{therm\;}\;$is the thermal vibrational energy of the reagents (0.018 eV at *T* = 300 K), $E_{µVTS}^{vib}\left(hv\right)$ is the vibrational energy in the reactive complex coming from photoionization excitation of the methanol, $E_{CH3OH^{+}}^{therm}$ is the thermal vibrational energy of the methanol cation (0.017 eV at *T* = 300 K), and $E_{CH3OH^{+}}^{vib}\left(hv\right)$ is the vibrational energy in the methanol coming from photoionization excitation. The variable *x* is the fraction of the energy *E_µVTS_* that goes into the vibrational degrees of freedom of the reactive complex, excluding the fraction (1 − *x*) that goes into the reactive coordinate. Hence, *xE_µVTS_* in Equation (2) represents the energy content of the vibrational levels of the complex in the VTS geometry, whereas (1 − *x*) *E_µVTS_* is the part of the reaction energy that becomes the relative kinetic energy of the two products. To calculate the fraction *x*, the rate coefficient of Equation (2) was set as equal to the experimental rate coefficient at the ionization threshold, where both $E_{µVTS}^{vib}\left(hv\right)$ and $E_{CH3OH^{+}}^{vib}\left(hv\right)\;$are zero. The value of *x* thus obtained is 0.66, which indicates that about ⅔ of the energy released during the reaction from the reagents to the µ*VTS* complex was kept inside its vibrations, whereas ⅓ of this energy went to the reactive coordinate.

**Figure 7:**
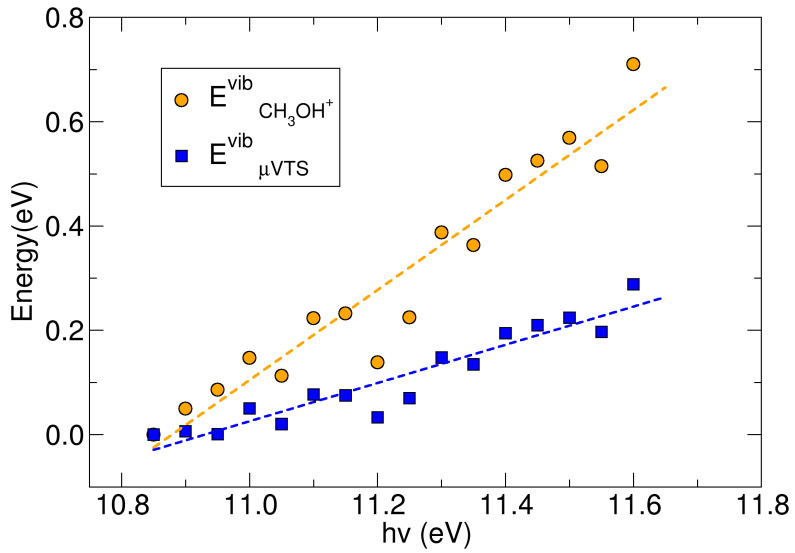
Vibrational energy of CH_3_OH^+^ and the µ*VTS* complex obtained by fitting the microcanonical rate with the experimental rates. Dashed lines are only drawn as a guide for the eye. See further details in the main text.

The $E_{µVTS}^{vib}\left(hv\right)$ and $E_{CH3OH^{+}}^{vib}\left(hv\right)$ at *hν* > EI_(CH3OH)_ were determined by fitting Equation (2) with the experimental rate coefficient at each *hν*. The results are reported in Figure 7, which shows that $E_{CH3OH^{+}}^{vib}\left(hv\right)$ has almost a linear dependence on ionization energy. At each *hν* the methanol cation always took more than 50% of the excitation energy (*E_exc_* = *hν* − EI), and at *hν* = 11.6 eV it reached about 0.7 eV, i.e., about 90% of the *E_exc_*, while the ejected electron took only 10% of this excitation energy. This behavior is consistent with the relatively floppy nature of the methanol cation, with many vibrational degrees of freedom able to absorb *E_exc_* above the ionization threshold. By contrast, $E_{µVTS}^{vib}\left(hv\right)$ had a slower dependence on *hν*, indicating that only a fraction of the vibrational energy of CH_3_OH^+^ was stored in the vibrational levels of the µ*VTS* complex, whereas the greater part of the vibrational energy of the methanol cation went into the reactive coordinate. Overall, the behavior of both $E_{µVTS}^{vib}\left(hv\right)\;$and $E_{CH3OH^{+}}^{vib}\left(hv\right)$ was strongly non-thermal, indicating that the reaction proceeds with energy fluxes driven by dynamical factors rather than thermal statistical mechanisms.

In conclusion, this work provides rate coefficients and energetic details that can be used in the chemical networks employed in astro- and atmospheric chemistry. The reaction mechanism explored in this study pertains to methane and methanol, which are complex organic molecules (COMs) present in the interstellar medium. The search for these molecules in space has been significantly enhanced by the advent of the Mid-InfraRed Instrument (MIRI) on the James Webb Space Telescope (JWST), which is unveiling the chemistry of extraterrestrial species with unprecedented spectral resolution and sensitivity [51].

## 4. Conclusions

The reaction studied in the present work is of considerable interest due to the nature of the reactants and the products. Both are involved in processes relevant from the point of view of atmospheric chemistry and astrochemistry. The combination of experimental investigation with synchrotron radiation and a theoretical approach based on mixed thermal/non-thermal rate coefficient formulation is well suited for the study of non-equilibrium reaction mechanisms. The non-thermal nature of the reaction appears evident when the methanol cation reagent is prepared with an excess of vibrational energy following the absorption of a photon above the ionization threshold. In this case, the subtle game between the energy flows directed towards the vibrational degrees of freedom of the VTS complex and the reaction coordinate determines a rate coefficient that decreases as the energy of the ionizing photon increases. This can have significant implications in the low-density ISM, where photons beyond the ionization threshold can slow down the reaction. Contrarily, the reaction can be significantly accelerated in thermal equilibrium environments, as for instance at the boundary between the troposphere and the stratosphere, where, as the temperature decreases, the calculation of the canonical rate coefficient shows that the reaction can already reach its maximum value imposed by capture theory at around 200 K. This behavior could have important consequences in modifying the chemical budget following the presence of ionizing agents such as cosmic rays. The products of the reaction, the methyl radical and protonated methanol, can then in turn start a network of chemical reactions capable of influencing the balance of species present in the environment considered.

## Figures and Tables

**Table 1:** Parameters for the fitting of the experimental rate coefficients as a function of the photon energy (*hν*) and of the canonical rate as a function of temperature (*T*).

	Exp Rate Coefficient ^(1)^ (P = 5.5·10^−5^ mbar)	Canonical Rate Coefficient ^(2)^
α (cm^3^ molecule^−1^s^−1^)	3.006·10^−10^	2.761·10^−11^
β	−6.277	1.261
γ	−7.495 eV	−913.839 K

^(1)^ *k*(*hν*) = α(*hν*/10.85)^β^exp(−γ/*hν*); ^(2)^ *k*(*T*) = α(*T*/300)^β^exp(−γ/*T*).

## Data Availability

The data presented in this study are available in the article.
